# Cardiac MRI findings in patients with Myocarditis

**DOI:** 10.1186/1532-429X-13-S1-P323

**Published:** 2011-02-02

**Authors:** Darach O h-Ici, Yves Louvard, Thierry Unterseeh, Thomas Hovasse, Marie Claude Morice, Jérôme Garot

**Affiliations:** 1 Institut Cardiovasculaire Paris Sud, Massy, France

## Background

Cardiac Magnetic Resonance Imaging (CMR) is a . The recent conjoint report of the American College of Cardiology and the SCMR has classified its use as “appropriate” or “Class A Indication” in inflammatory cardiomyopathy. The aim of this study was to describe the typical findings in patients diagnosed with myocarditis undergoing CMR.

## Methods

Patients who underwent CMR at ICPS who were diagnosed with myocarditis on the basis of cardiac imaging and clinical findings (patients, n = 187) between November 2009 and September 2010 were identified through the use of the cardiology database. All patients underwent a similar protocol to look for:

1. LV and RV function

2. The presence of pleural effusion

2. Tissue edema, as seen by an elevated T2 signal

3. Myocardial necrosis or scarring, as indicated by the presence of late gadolinium enhancement (LGE)

## Results

Most patients (n=175, 93.6%) had LGE in a pattern sparing the subendocardium. Areas of hypokinesis were common (n = 62, 33.2%), as was the presence of myocardial edema (n = 58, 31.0%). Pericardial effusions were less common (n = 35, 18.7%). Of those patients who also underwent stress perfusion CMR, none showed evidence of ischemia. Left Ventricular ejection fractions were largely preserved (57.3% ± 10.4%) and end-diastolic volumes normal (69.2 ± 21.4 ml/m^2^).

## Conclusion

CMR abnormalities are common in patients with myocarditis. A typical pattern of delayed enhancement is by far the commonest abnormal finding in this group of patients. The use of a combination of CMR findings in keeping with the clinical picture is necessary for accurate diagnosis.

